# Increased remission with fewer corticosteroids and more biologics in rheumatoid arthritis at 7-year follow-up in real-life conditions

**DOI:** 10.1038/s41598-022-06584-y

**Published:** 2022-02-15

**Authors:** Guillaume Larid, Justine Vix, Ronan Garlantezec, Elodie Loppin, Elisabeth Gervais

**Affiliations:** 1 Rheumatology Department, University Hospital of Poitiers, CHU de Poitiers, 2 Rue de la Milétrie, 86021 Poitiers, France; 2 LITEC Laboratory, EA 4331, Poitiers University, Poitiers, France; 3 Epidemiology Department, University of Rennes, Rennes, France

**Keywords:** Rheumatoid arthritis, Prognosis

## Abstract

Remission in rheumatoid arthritis (RA) is an important therapeutic target that is not easy to achieve in real-life conditions. Some prognostic factors have been identified but the literature is variable. The objectives of this study were to evaluate the remission rate and the maintenance of remission in patients with RA over 7 years of follow-up in real-life conditions and to identify prognostic factors of long-term remission. Patients with RA seen at the Poitiers University Hospital were identified and clinical and biological data were collected. Data were analysed after 1 year and 7 years. Twice as many patients were in remission at 7 years than at 1 year of follow-up. 48.6% of patients who were not in remission at 1 year obtained remission at 7 years of follow-up. Patients achieving remission were more often receiving coprescription of csDMARDs and bDMARDs. Patients not in remission at 7 years were given more corticosteroids at higher doses. After 7 years of follow-up, low initial disease activity and use of csDMARDs and bDMARDs appeared to be independent positive predictive factors. Once obtained at one year, remission was maintained for 76% of our patients. As a conclusion, modern management of RA, whatever disease duration, leads to remission rates similar to those of early RA after 7 years of follow-up.

## Introduction

Remission in rheumatoid arthritis (RA) is an important therapeutic target^1^, as it is associated with better long-term physical function^2^ and is a way of achieving better productivity and lower costs for our society^3^. This target has been more frequently achieved since the advent of new therapies. Data on remission in RA are variable (up to 53%) as it depends on therapeutics, disease duration, duration of treatment and measure of disease activity^4^. However, when patients with “real life conditions” are involved, the remission level is under this expectation^5^.

As remission is a target in the treatment of RA, several authors have tried to determine whether prognostic factors exist. Unfortunately, the results in various studies differ and follow-up was short^2,6^.

Among prognosis factors, male sex, young age, short disease duration, lack of ACPA and rheumatoid factor have been identified^1^.

First mentioning of the concept of “treat-to-target” (T2T) in recommendations was found in the EULAR guidelines for the management of RA in 2010^7^. Since then, this strategy has been widely disseminated in rheumatologist’s daily practice.

The objective of this study was to evaluate the remission rate and the maintenance of remission in patients with RA after 7-years follow-up in “real life” conditions, during a period encompassing T2T emergence. The second objective was to define prognostic factors for long-term remission and to identify a potentially better treatment strategy leading to sustained remission.

## Patients and methods

### Patients

Patients with RA according to the American College of Rheumatology (ACR) criteria^8^ and seen in an outpatient clinic between January and December 2008 at the Rheumatology department of the University Hospital of Poitiers were identified through review of records. Clinical and biological data were analyzed after 1 year (in 2009) and after 7 years (2015).

### Evaluation

For the purposes of this retrospective monocentric observational study, « real-life » clinical data were collected. The demographic data collected included age at inclusion, sex, disease duration, rheumatoid factor (RF) and anti-citrullinated protein antibodies (ACPA) as detected by the anti-CCP ELISA assay method. Previous DMARD therapy was recorded. Disease activity was analyzed (tender joints, swollen joints, Overall Global Assessment (rated from 0 to 100 using a visual analogic scale), erythrocyte sedimentation rate (ESR), and C-reactive protein (CRP)). The disease activity score including 28 joints (DAS28)^9^ was calculated on the basis of these criteria at the time of the visit. Radiographic erosions on X-Rays were also noted. Use of corticosteroids, conventional DMARDs (csDMARDs) and biologics (bDMARDs) was collected at inclusion and at 1 and 7-year follow-up. Disease activity measured by the DAS28-ESR was collected at inclusion and at 1-year (2009) and 7-year follow-up (2015). Remission was defined as DAS28 < 2.6, low disease activity (LDA) as DAS28 ≤ 3.2 and high disease activity (HDA) as DAS28 > 5.1^10^. When only CRP was available, ESR DAS 28 was calculated with the following formula: ESR DAS_28_ = 1.01 × DAS_28CRP_ + 0.590^11^. Remission maintenance was defined by remission in 2009 and 2015 with the same treatment.

### Statistical analysis

Qualitative data were expressed as percentages and quantitative data as means ± standard deviations. Univariate analysis was conducted using Student test (or Wilcoxon, as appropriate) for quantitative data and Chi^2^ for qualitative data. Multivariable logistic regression was performed to study the characteristics associated with remission in 2015. Variables tested in association with remission were age at inclusion, csDMARDs, bDMARDs, sex, RF, ACPA, erosions, initial DAS 28 score. The association was presented as odds ratio (OR) with 95% confidence intervals (95% CI). A p value at 0.05 was considered as significant. Statistical analysis was performed using SAS software, version 9.1 (SAS Institute Inc., Cary, NC, USA).

### Ethics

The study was approved by the local institutional ethics committee of University Hospital of Poitiers and conducted in accordance with the Declaration of Helsinki. An oral consent was obtained from all the participants. Written consent was not required according to the MR-004 French legislation.

## Results

### Patient characteristics

Three hundred and sixty-four patients were seen in a University Hospital Rheumatology department between 1st January 2008 and 1st December 2008.

**Figure 1:**
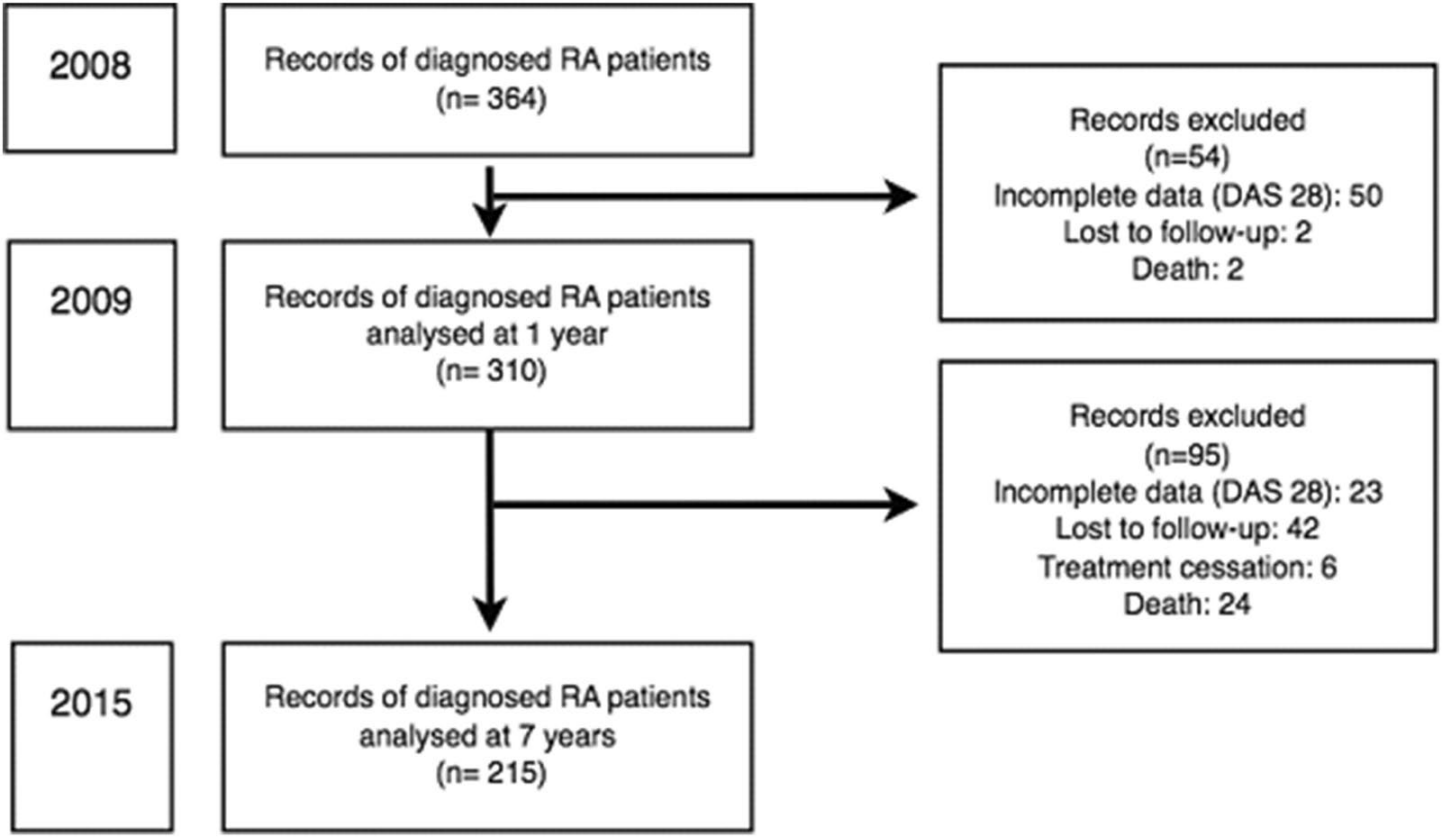
Flowchart.

Among the 364 records of RA patients identified, 310 were analyzed in 2009 and 215 in 2015. Patients were excluded (54 in 2009 and 95 in 2015) in the event of incomplete data, treatment cessation, loss to follow-up or death (Fig. 1).

Patients followed until 2015 were significantly younger and more frequently ACPA-positive than patients loss to follow-up (Table 1). In terms of activity, they were less in remission and more in moderate disease activity (Table 1). In terms of treatment, they were given more corticosteroids, less biologics, especially in monotherapy, and were more often without DMARDs at all (Supplementary Table 3). Detailed comparisons between the different groups are described in Supplementary Tables 1–3.

**Table 1:** Patient characteristics: patients consulting with a Rheumatoid Arthritis in Rheumatology department at the Poitiers University Hospital in 2008 according to their inclusion or not in follow-up.

Patient characteristics	Patients followed up until 2015 (n = 215)	Patients lost to follow-up (n = 149)	P	
Female sex	166 (77%)	120 (80%)	0.45	
Mean age (years )	60.4 (33–98)	67.1 (27–89)	** < 0.0001**	
Disease duration (years)	19.2 (7, 3–55)	15 (1–60)	0.19	
RF positivity	165 (77%)	117 (79%)	0.69	
ACPA positivity	157 (73%)	72 (48%)	** < 0.0001**	
Erosions	191 (89%)	141 (95%)	0.06	
**Initial DAS28**	(n = 215)	(n = 113)		
DAS 28 < 2.6	69 (32.1%)	22 (19.5%)	**0.015**	**0.013**
2.6 < DAS 28 < 3.2	42 (19.5%)	15 (13.3%)	0.155	
3.2 < DAS 28 < 5.1	83 (38.6%)	62 (54%)	**0.005**	
DAS 28 > 5.1	21 (9.8%)	14 (12.4%)	0.465	

*RF* rheumatoid factors, *ACPA* anti-citrullinated peptide antibodies. Significant values are in bold.

### RA activity in “real-life” conditions

There was a significant decrease of DAS28 in the patients with RA seen in 2015 compared to those seen in 2009 (mean difference 2015–2009 = −0.58 CI95% [− 0.77; − 0.40]). There were more patients with LDA (53.7% in 2009 versus 71.1% in 2015) and almost twice as many patients were in remission in 2015 compared to 2009.

**Figure 2:**
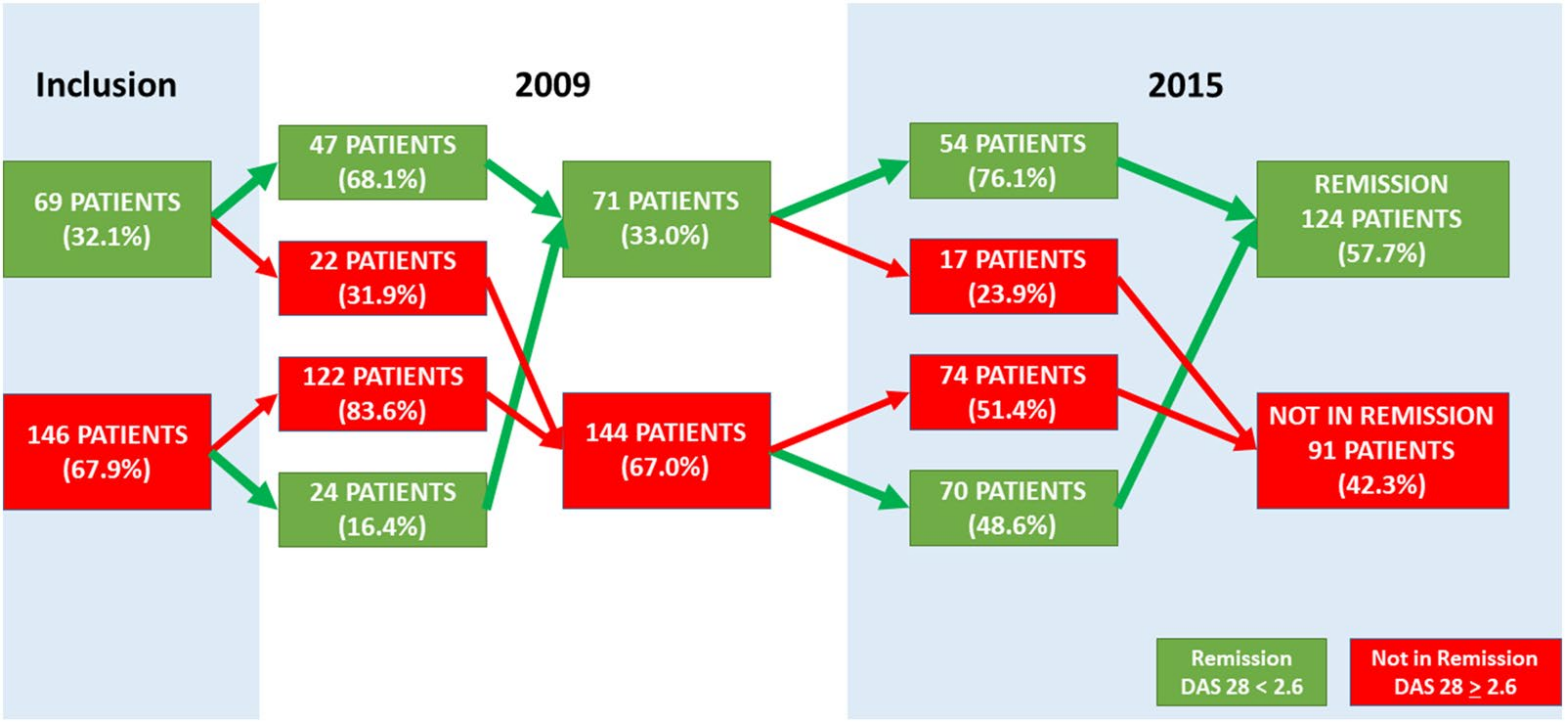
Description of remission and remission maintenance between 2009 and 2015: Patients (n = 215) consulting with Rheumatoid Arthritis in Rheumatology department at the Poitiers University Hospital in 2008 and follow-up until 2015.

Among the 71 patients in remission in 2009, 76% (n = 54) were still in (long-term) remission in 2015. Seventy (48.6%) out of the 144 patients with DAS 28 > 2.6 in 2009 were in remission in 2015. During the 7 years of follow-up, 70 patients (32.6%) who had an active disease at 1 year of follow-up went into remission. On the other hand, between 2009 and 2015, 17 patients (7.9%) went out of remission. All in all, remission was achieved for 124 patients (57.7%) (Fig. 2).

### Comparison of RA treatments in 2009 and 2015 (Table 2)

During the 7-year follow-up, there was increased prescription of DMARDs, particularly biologics and a significant decrease of patients on corticosteroids (p < 0.0001): 56% of patients with an average of 7.31 mg per day in 2008 compared to 23.7% with an average of 6.7 mg per day in 2015. As regards DMARDs, biologics were more frequently prescribed to patients in 2015 (51.1% in 2015 vs 30% in 2009, p < 0.0001). As regards csDMARD and bDMARD coprescription, there was no significant increase in 2015 compared with 2009 (55/215 in 2009 vs 63/215 in 2015; p = 0.387). However, there was a significant increase of bDMARD monotherapy (24/215 in 2009 vs 47/215 in 2015; p = 0.0028).

**Table 2:** Rheumatoid arthritis treatment prescribed in patient follow-up until 2015 (n = 215).

	In 2009	In 2015	P
Corticosteroids	120 (55.8%)	57 (23.7%)	** < 0.0001**
Dose of corticosteroids	7.31 mg/day	6.7 mg/day	0.12
csDMARDs only	118 (54.9%)	97 (45.1%)	**0.0035**
Biologics	79 (36.7%)	110 (51.1%)	**0.0035**
csDMARD and bDMARD coprescription	55 (25.6%)	63 (29.3%)	0.586
bDMARDs only	24 (11.2%)	47 (21.9%)	**0.004**
No DMARD	18 (8.4%)	8 (3.7%)	0.07

Significant values are in bold.

### Comparison of treatment use in 2015 between patients in remission and those not in remission (Table 3)

Treatments prescribed in patients in 2015 have been analyzed. Patients achieving remission were significantly more often receiving bDMARDs (p < 0.001), especially TNF-alpha inhibitors (p = 0.006). They were also significantly more often receiving csDMARD and bDMARD coprescription (p = 0.02). Patients not in remission were given more corticosteroids (p = 0.02) at higher doses (p = 0.013). Coprescription of csDMARDs and bDMARDs was significantly associated with remission compared with csDMARD monotherapy (44/63 in remission with combined therapy vs 50/97 with csDMARDs only; OR = 2.177 [1.113–4.315]; p = 0.022) but not when compared with bDMARD monotherapy (44/63 in remission with combined therapy vs 29/47 with bDMARDs only; OR = 1.437 [0.6541–3.056]; p = 0.371).

**Table 3:** Comparison of treatment use in 2015 between patients in remission and those not in remission (n = 215).

	Patients in remission in 2015 (n = 124)	Patients not in remission in 2015 (n = 91)	P
Number of DMARDs (min–max)	2 (1–6)	3 (0–7)	**0.01**
Corticosteroids	23 (18.5%)	30 (32.9%)	**0.02**
Corticosteroid dose (mg; mean)	6.59	7.05	**0.013**
No DMARD	1 (0.8%)	7 (7.7%)	**0.011**
csDMARDs only	50 (40.3%)	47 (51.6%)	0.13
bDMARDs	73 (58.8%)	37 (40.7%)	** < 0.001**
csDMARDs + bDMARDs	44 (35.4%)	19 (20.9%)	**0.02**
bDMARDs only	29 (23.4%)	18 (19.8%)	0.63
**bDMARDs type**			
TNF inhibitors	44 (35.4%)	17(18.6%)	**0.006**
Etanercept	25 (20.0%)	9 (9.8%)	**0.04**
Adalimumab	16 (12.9%)	2 (2.2%)	**0.005**
Infliximab	1	5 (5.5%)	–
Golimumab	1	1	–
Certolizumab	1	0	–
Abatacept	9 (7.25%)	9 (9.89%)	0.49
Tocilizumab	13 (10.4%)	6 (7.2%)	0.32
Rituximab	6 (4.8%)	5(5.5%)	0.83
Anakinra	1	0	–

Significant values are in bold.

### Analysis of remission maintenance predictive factors (Table 4)

After 7-year follow up, low initial disease activity, use of csDMARDs, and use of bDMARDs for treatment of RA appeared to be independent positive predictive factors. Moreover, patients without ACPA were more likely to be in remission, but this was not statistically significant in the multivariable analysis. Age at inclusion, sex, rheumatoid factor positivity, and presence of erosions were not statistically significant.

**Table 4:** Multivariable analysis of remission maintenance predictive factors (logistic regression) (n = 215).

	Odd-ratio	CI	p*-*value
Age at inclusion	0.9801	0.9488–1.011	0.2102
csDMARDs	4.079	1.416–12.82	**0.0117**
bDMARDs	2.782	1.068–7.713	**0.0411**
Male sex	1.682	0.6529–4.571	0.2906
Rheumatoid factor positivity	2.570	0.9259–7.559	0.0756
ACPA positivity	0.4220	0.1446–1.163	0.1020
Erosions	0.6295	0.2285–1.642	0.3531
DAS28 at inclusion	0.6412	0.4385–0.9188	**0.0178**

*ACPA* anti-citrullinated peptide antibodies. Significant values are in bold.

## Discussion

This study analyzed clinical outcomes on 7 years of follow-up of RA patients in “real-life conditions”. Remission was obtained for 33% of patients at 1 year (in 2009) and increased (almost doubled) up to 57.7% at 7 years (in 2015). These results concord with those of the Australian OPAL Cohort showing a significant improvement in disease activity over 5 years of follow up (remission rate at 36.7% in 2009 increased to 53.5% in 2014)^12^ and other studies that found long-term remission levels of 55.5%, 53% and 55.5%^13–15^. Furthermore, our results demonstrate that patients being in remission at the 1-year follow-up visit had a greater chance of still being in remission at the 7-year follow-up visit, with 76% of them in long-term sustained remission. This is concordant with the results of a subgroup of the ESPOIR cohort in which the remission rate, during the 5 years of follow-up, ranged from 77.8% to 81% at the different yearly visits in patients who achieved DAS28 remission at both the 6- month and the 12-month visits^16^.

In our study, patients were not included at a particular moment in their disease course so we analysed here a fairly arbitrary 7 years in their disease course. However, including the patients in 2008 allow us to evaluate the impact of the dissemination of the concept of “treat-to-target” (T2T) in the rheumatologists daily practice, this strategy being widely disseminated until this time, the crucial time point being its first mentioning in recommendations in the EULAR guidelines for the management of RA in 2010^7^. Therefore it seems that, even if it’s well known that early remission in early RA is the better way to achieve sustained remission as detailed in the review of Monti et al.^17^, targeting remission at any time points could lead to similar remission rates after 7 years than in early RA studies, whatever disease duration.

Impact of T2T strategy implementation in rheumatologists’ daily practice on those results is likely since studies made before adding T2T in RA management guidelines showed really low levels of sustained remission of 15–20% at 7 years using DAS28^18–20^.

In this long-standing “real life conditions” study, there was a significant decrease of corticosteroid use over time. This is concordant with other publications^21^ explaining that even if patients have a high dose of corticosteroid at treatment initiation, fewer keep it on the long term^13^.

As regards DMARDs, bDMARDs were more frequently prescribed to patients in 2015, similarly to recent data^13,15^. As in the NOR-DMARD cohort, co-prescription of conventional DMARDs and biologics provided a better chance to achieve sustained remission than csDMARDs monotherapy^6,21–23^. When comparing patients in remission with those who did not achieve remission in 2015, there was a significant difference in the prescriptions of bDMARDs, especially etanercept and adalimumab. However, the availability of other biologics was not the same in 2008, when the patients were included. Even if a difference in remission influenced by the bDMARD used was found, results should be evaluated with caution because of the monocentric recruitment leading to a relatively small number of patients in the subgroup.

Combined therapy or bDMARDs alone was equivalent to csDMARDs alone at 1 year, but not at 7 years, where association of csDMARDs and bDMARDs was more associated with remission than csDMARDs alone. In our study, association of csDMARDs and bDMARDs was not statistically different from bDMARDs alone. It has been reported in multiple systematic literature review^23–26^ that coprescription of csDMARDs and bDMARDs are more efficient than bDMARD monotherapy. In our study, most patients on bDMARD monotherapy where treated by etanercept or tocilizumab which are licensed for monotherapy in RA treatment. The absence of statistical difference observed in our study is probably a consequence of the low number of patients receiving these treatments.

Multivariable analysis found low inclusion disease activity (DAS 28 score), and treatment by csDMARDs and bDMARDs as predictive factors of achieving sustained remission, this finding was concordant with previous studies^4,5,27^. Sex and presence of rheumatoid factors were associated with prognosis only in univariate analysis. Some factors associated with remission in other publications^28^ could not be analyzed because of missing data at inclusion (family history of RA or autoimmune disease, presence of HLA-DRB1 shared epitope, smoking habits, overall global assessment, body mass index).

The strengths of this study are length of follow-up and the remission rate obtained after 7 years of follow up in “real-life conditions”. Indeed, it has been demonstrated in the literature that less than 10% of daily practice RA patients satisfy criteria for participation in randomized controlled trials^29^, making real world data more relevant to extrapolate results of effectiveness in daily practice.

The weakness of this study is the DAS 28 score measure. It can overestimate the number of patients in remission compared to more stringent criteria (ACR-EULAR Boolean, SDAI or CDAI)^30^. However, it can be different in “real-life conditions” and studies have shown that DAS28 monitoring in patient follow up is not associated with poorer clinical or structural outcomes^20,31^.

Another limit of the study is a bias related to the large number of patients who didn’t complete the 7-year follow-up. Reasons for people not completing the study were incomplete data record for 73 of them, loss to follow-up for 44 of them, treatment cessation for 6 of them, and death for 26 of them. Patients were followed in usual care setting therefore were not included in a prospective study with mandatory appointment which can explain the number of patients lost to follow-up. When comparing both groups, we’ve found that people lost to follow-up were older and with less ACPA positivity. They were also given more corticosteroids and less biologics with more patients without any DMARD. This could be, partly explained by patients being older with more comorbidities which were often the reasons to not initiate bDMARDs or to maintain patients only under corticosteroids as seen in the medical records. Moreover, our population is only representative of RA patients followed-up in a university hospital, which could be somehow different of patients only seen by liberal rheumatologists.

In conclusion, during this 7-year follow-up, a large number of patients achieved strict DAS28 remission with fewer corticosteroids and more biologics. Once obtained at one year, remission was maintained in 76% of our patients between 2009 and 2015. Even if previous management of patients with RA was not aiming for remission, modern management of RA at any time in the disease course could lead to similar remission rates after 7 years than in early RA studies, whatever disease duration.

## Supplementary Information

Supplementary Tables.

## Data Availability

The datasets generated during and/or analyzed during the current study are available from the corresponding author on reasonable request.

## References

[1] Smolen JS (2020). EULAR recommendations for the management of rheumatoid arthritis with synthetic and biological disease-modifying antirheumatic drugs: 2019 update. Ann. Rheum. Dis..

[2] Castrejón I (2016). Prediction of remission in a French Early Arthritis Cohort by RAPID3 and other core data set measures, but not by the absence of rheumatoid factor, anticitrullinated protein antibodies, or radiographic erosions. J. Rheumatol..

[3] Radner H, Smolen JS, Aletaha D (2014). Remission in rheumatoid arthritis: benefit over low disease activity in patient-reported outcomes and costs. Arthritis Res. Ther..

[4] Katchamart W (2010). Predictors for remission in rheumatoid arthritis patients: A systematic review. Arthritis Care Res..

[5] Acosta-Mérida Á, Naranjo A, Rodríguez-Lozano C (2020). Prognostic factors for sustained remission in a “real life” cohort of rheumatoid arthritis patients. Reumatología Clínica.

[6] Goekoop-Ruiterman YPM (2008). Clinical and radiographic outcomes of four different treatment strategies in patients with early rheumatoid arthritis (the BeSt study): A randomized, controlled trial. Arthritis Rheum..

[7] Smolen JS (2010). EULAR recommendations for the management of rheumatoid arthritis with synthetic and biological disease-modifying antirheumatic drugs. Ann. Rheum. Dis..

[8] Aletaha D (2010). 2010 Rheumatoid arthritis classification criteria: An American College of Rheumatology/European League Against Rheumatism collaborative initiative. Arthritis Rheum..

[9] Prevoo ML (1993). Validity and reliability of joint indices. A longitudinal study in patients with recent onset rheumatoid arthritis. Br. J. Rheumatol..

[10] van Riel PL, van Gestel AM (2000). Clinical outcome measures in rheumatoid arthritis. Ann. Rheum. Dis..

[11] Inoue E, Yamanaka H, Hara M, Tomatsu T, Kamatani N (2007). Comparison of Disease Activity Score (DAS)28-erythrocyte sedimentation rate and DAS28-C-reactive protein threshold values. Ann. Rheum. Dis..

[12] Littlejohn G (2015). Patients with rheumatoid arthritis in the Australian OPAL Cohort show significant improvement in disease activity over 5 years: A multicenter observational study. J. Rheumatol..

[13] Minichiello E, Semerano L, Boissier M-C (2016). Time trends in the incidence, prevalence, and severity of rheumatoid arthritis: A systematic literature review. Joint Bone Spine.

[14] Markusse IM (2016). Long-Term Outcomes of Patients With Recent-Onset Rheumatoid Arthritis After 10 Years of Tight Controlled Treatment: A Randomized Trial. Ann Intern Med.

[15] Haugeberg G, Hansen IJW, Soldal DM, Sokka TT (2015). years of change in clinical disease status and treatment in rheumatoid arthritis: Results based on standardized monitoring of patients in an ordinary outpatient clinic in southern Norway. Arthritis Res. Ther..

[16] Combe B (2015). Comparison of the long-term outcome for patients with rheumatoid arthritis with persistent moderate disease activity or disease remission during the first year after diagnosis: Data from the ESPOIR cohort. Ann. Rheum. Dis..

[17] Monti S, Montecucco C, Bugatti S, Caporali R (2015). Rheumatoid arthritis treatment: the earlier the better to prevent joint damage. RMD Open.

[18] Einarsson JT, Geborek P, Saxne T, Kristensen LE, Kapetanovic MC (2016). Sustained remission improves physical function in patients with established rheumatoid arthritis, and should be a treatment goal: A prospective observational cohort study from Southern Sweden. J Rheumatol.

[19] Prince FH (2012). Sustained rheumatoid arthritis remission is uncommon in clinical practice. Arthritis Res. Ther..

[20] Svensson B (2013). Long-term sustained remission in a cohort study of patients with rheumatoid arthritis: Choice of remission criteria. BMJ Open.

[21] Aga A-B (2015). Time trends in disease activity, response and remission rates in rheumatoid arthritis during the past decade: Results from the NOR-DMARD study 2000–2010. Ann. Rheum. Dis..

[22] Uhlig T (2016). Achievement of remission and low disease activity definitions in patients with rheumatoid arthritis in clinical practice: Results from the NOR-DMARD study. J. Rheumatol..

[23] Emery P (2018). Efficacy of monotherapy with biologics and JAK inhibitors for the treatment of rheumatoid arthritis: A systematic review. Adv. Ther..

[24] Nam JL (2014). Efficacy of biological disease-modifying antirheumatic drugs: A systematic literature review informing the 2013 update of the EULAR recommendations for the management of rheumatoid arthritis. Ann. Rheum. Dis..

[25] Nam JL (2017). Efficacy of biological disease-modifying antirheumatic drugs: A systematic literature review informing the 2016 update of the EULAR recommendations for the management of rheumatoid arthritis. Ann. Rheum. Dis..

[26] Nam JL (2010). Current evidence for the management of rheumatoid arthritis with biological disease-modifying antirheumatic drugs: A systematic literature review informing the EULAR recommendations for the management of RA. Ann. Rheum. Dis..

[27] Fornaro M, Cacciapaglia F, Lopalco G, Venerito V, Iannone F (2021). Predictors of long-term clinical remission in rheumatoid arthritis. Eur. J. Clin. Invest..

[28] Quintana-Duque MA (2016). Predictors of remission, erosive disease and radiographic progression in a Colombian cohort of early onset rheumatoid arthritis: A 3-year follow-up study. Clin. Rheumatol..

[29] Vashisht P, Sayles H, Cannella AC, Mikuls TR, Michaud K (2016). Generalizability of patients with rheumatoid arthritis in biologic agent clinical trials: RA RCT generalizability. Arthritis Care Res..

[30] Mierau M (2007). Assessing remission in clinical practice. Rheumatology (Oxford).

[31] Steunebrink LMM (2016). Recently diagnosed rheumatoid arthritis patients benefit from a treat-to-target strategy: Results from the DREAM registry. Clin. Rheumatol..

