# Supplementary material for: Increased remission with fewer corticosteroids and more biologics in rheumatoid arthritis at 7-year follow-up in real-life conditions
Source: Sci Rep. 2022 Feb 15;12:2563. doi: 10.1038/s41598-022-06584-y (PMC8847581; doi:10.1038/s41598-022-06584-y)
Supplement: Supplementary file 1 — Supplementary Tables. [file 41598_2022_6584_MOESM1_ESM.docx]

**Supplementary tables**

Increased remission with fewer corticosteroids and more biologics in Rheumatoid Arthritis at 7-year follow-up in real-life conditions

Guillaume LARID, MD(1,2) (ORCID ID : 0000-0003-2317-2852), Justine VIX, MD (1), Ronan GARLANTEZEC, MD, PhD (3), Elodie LOPPIN, MD (1), Elisabeth GERVAIS*, MD, PhD (1,2) (ORCID iD : 0000-0003-3513-2989)

1. Rheumatology Department, University Hospital of Poitiers, FRANCE
2. LITEC laboratory, EA 4331, Poitiers University, France

(3) Epidemiology Department, University of Rennes, France

Supplementary Table 1 : Comparison of disease activity at inclusion between the whole initial population and the complete follow-up population

|  | **Whole population with DAS28**  **(n = 328)** | **Complete follow-up population**  **(n = 215)** | **p** | **p** |
| --- | --- | --- | --- | --- |
| **DAS 28 < 2.6** | 91 (27.7%) | 69 (32.1%) | 0.277 | 0.524 |
| **2.6 < DAS 28 < 3.2** | 57 (17.4%) | 42 (19.5%) | 0.524 |  |
| **3.2 < DAS 28 < 5.1** | 145 (44.2%) | 83 (38.6%) | 0.196 |  |
| **DAS 28 > 5.1** | 35 (10.7%) | 21 (9.8%) | 0.735 |  |

Chi square test

Supplementary Table 2 : Comparison of treatment at inclusion between the whole initial population and the complete follow-up population

|  | **Whole population**  **(n=364)** | **Complete follow-up population (n=215)** | **P** |
| --- | --- | --- | --- |
| **Corticosteroids** | 187 (51.4%) | 114 (53%) | 0.70 |
| **Dose of corticosteroids**  **(mg/day ; mean)** | 7.3 | 6.32 | 0.12 |
| **csDMARDs only** | 221 (60.7%) | 122 (56.7%) | 0.88 |
| **Biologics** | 99 (27.2%) | 74 (34.4%) | 0.07 |
| **csDMARD and bDMARD coprescription** | 70 (19.5%) | 45 (20.9%) | 0.62 |
| **bDMARDs only** | 29 (7.7%) | 29 (13.5%) | **0.03** |
| **No DMARD** | 44 (12.1%) | 19 (8.8%) | 0.22 |

Chi square test and Wilcoxon test

Supplementary Table 3 : Comparison of treatment at inclusion between the lost to follow-up group with DAS28 available and the complete follow-up population

|  | **Lost to follow-up population**  **(n=149)** | **Complete follow-up population (n=215)** | **p** |
| --- | --- | --- | --- |
| **Corticosteroids** | 73 (49%) | 114 (53%) | 0.45 |
| **Dose of corticosteroids** | 8.28 | 6.32 | **< 0.001** |
| **csDMARDs only** | 99 (66.4%) | 122 (56.7%) | 0.06 |
| **Biologics** | 25 (16.8%) | 74 (34.4%) | **< 0.001** |
| **csDMARD and bDMARD coprescription** | 25 (16.8%) | 45 (20.9%) | 0.32 |
| **bDMARDs only** | 0 (0%) | 29 (13.5%) | **0.04** |
| **No DMARD** | 25 (16.8%) | 19 (8.8%) | **0.02** |

Chi square test and Wilcoxon test
